# Effects of Executive Function Training on Attentional, Behavioral and Emotional Functioning and Self-Perceived Competence in Very Preterm Children: A Randomized Controlled Trial

**DOI:** 10.3389/fpsyg.2019.02100

**Published:** 2019-09-13

**Authors:** Carolien A. van Houdt, Cornelieke S. H. Aarnoudse-Moens, Aleid G. van Wassenaer-Leemhuis, A. R. Céleste Laarman, Corine Koopman-Esseboom, Anton H. van Kaam, Jaap Oosterlaan

**Affiliations:** ^1^Department of Neonatology, Emma Children’s Hospital, Amsterdam UMC, University of Amsterdam, Amsterdam, Netherlands; ^2^Emma Neuroscience Group, Emma Children’s Hospital, Amsterdam UMC, University of Amsterdam, Amsterdam, Netherlands; ^3^Section of Clinical Neuropsychology, Vrije Universiteit Amsterdam, Amsterdam, Netherlands; ^4^Psychosocial Department, Emma Children’s Hospital, Amsterdam UMC, University of Amsterdam, Amsterdam, Netherlands; ^5^Department of Neonatology, Emma Children’s Hospital, Amsterdam UMC, Vrije Universiteit Amsterdam, Amsterdam, Netherlands; ^6^Department of Neonatology, University Medical Center Utrecht, Utrecht, Netherlands; ^7^Department of Pediatrics, Amsterdam Reproduction and Development, Emma Neuroscience Group, Emma Children’s Hospital, Amsterdam UMC, University of Amsterdam, Amsterdam, Netherlands

**Keywords:** intervention, premature, EF training, computerized, executive functions

## Abstract

**Objective:**

Very preterm children have poorer attentional, behavioral and emotional functioning than term-born children. Problems on these domains have been linked to poorer executive function (EF). This study examined effects of a game-formatted, comprehensive EF training on attentional, behavioral and emotional functioning and self-perceived competence in very preterm children.

**Study Design:**

Eighty-five children participated in a multi-center, double-blind, placebo and waitlist-controlled randomized trial. Children were recruited from neonatal follow-up units of two academic medical centers in The Netherlands. Eligible for inclusion were 8–12 year old children born very preterm (<30 weeks of gestation) and/or with extremely low birthweight (<1000 g) with parent reported attention problems. Children were randomly assigned to one of three treatment arms: EF training, placebo training or waitlist. The EF and placebo training involved a 6 weeks, 25 (30–45 min) sessions training program. Attentional functioning (Attention Network Test), behavioral and emotional functioning (parent and teacher Strengths and Difficulties questionnaire) and self-perceived competence (Self-Perception Profile for Children) were assessed at baseline, at the end of the training program and 5 months after the training was finished. Data analyses involved linear mixed model analyses.

**Results:**

Children in the EF training arm significantly improved on all training tasks over the course of the EF training program. Despite these improvements on the EF training tasks, there were no significant differences over time on any of the outcome measures between the three treatment arms, indicating that this computerized EF training program had no beneficial effects.

**Conclusion:**

Although there were significant improvements in the EF training tasks, there was no generalization of these improvements to any of the outcome measures. Thus, our findings do not support the use of computerized EF training programs. Future research should investigate effectivity of more ecologically valid, real-world like EF training programs.

## Introduction

Between 0.7 and 1.4% of all live born children in Western countries are born very preterm (gestational age [GA] < 32 weeks) (Delnord et al., 2017). Long-term consequences of very preterm birth have been intensively investigated in the domains of cognitive, academic, behavioral and emotional functioning, with very preterm children showing substantial problems in all of these domains (Bhutta et al., 2002; Anderson et al., 2003; Aarnoudse-Moens et al., 2009; Mulder et al., 2009; Blencowe et al., 2013; Aylward, 2014; Ritchie et al., 2015; Allotey et al., 2017; Twilhaar et al., 2017). For example, executive functions (EF), which is an umbrella term for a set of higher-order cognitive functions allowing for top–down, goal-directed behavior, are adversely affected in very preterm children (Aarnoudse-Moens et al., 2009; Mulder et al., 2009; van Houdt et al., 2019). Deficits in EF have been shown to play an important underlying role in both the academic as well as the behavioral and emotional functioning problems that very preterm children encounter (Nadeau et al., 2001; Taylor et al., 2006; Mulder et al., 2010, 2011; de Kieviet et al., 2012; Loe et al., 2012; Aarnoudse-Moens et al., 2013; Alduncin et al., 2014). For example, EF performance has been shown to predict math performance in very preterm children at primary school (Aarnoudse-Moens et al., 2013) and working memory has been shown to account for academic attainment (Mulder et al., 2010). Furthermore, working memory has been shown to account for attention problems in very preterm children at school-age (Nadeau et al., 2001; Mulder et al., 2011; de Kieviet et al., 2012). Last, poorer EF performance has been shown associated with poorer social competence in very preterm children at preschool age (Alduncin et al., 2014) and school-age (Taylor et al., 2006; Loe et al., 2012).

In the past decade, an increasing number of studies have addressed the efficacy of computerized interventions to improve EF, with Cogmed Working Memory Training (CWMT) (Klingberg et al., 2005) being the most widely studied computerized EF training program. CWMT for school-age children involves gamified verbal and visuospatial working memory training tasks presented on a space-themed interface design. Children’s scores are presented on the screen to challenge children to outperform their own scores and difficulty level is automatically adjusted according to the child’s performance. CWMT is played five times a week for 30–45 min per session. Studies on CWMT in children with Attention Deficit/Hyperactivity Disorder (ADHD) have shown promising results in improving working memory and also reported some promising transfer effects to untrained functions (Klingberg et al., 2005; Beck et al., 2010; Green et al., 2012; Hovik et al., 2013; Chacko et al., 2014). Compared to a wait-list control group, CWMT was reported to improve verbal and non-verbal working memory storage, visuospatial working memory, verbal working memory, parent-rated working memory and parent-rated inattention symptoms (Beck et al., 2010; Hovik et al., 2013). Furthermore, compared to a placebo control group, CWMT was reported to improve trained working memory tasks and untrained performance on tasks assessing visuospatial working memory, verbal working memory, response inhibition and complex reasoning. Furthermore beneficial effects have been reported on parent-rated inattention and hyperactivity/impulsivity symptoms and on observed behaviors during an academic task (Klingberg et al., 2005; Green et al., 2012; Chacko et al., 2014). There is also some evidence of neural changes following CWMT and associations between these neural changes and improved working memory, both in healthy children and adults (Barnes et al., 2016; Metzler-Baddeley et al., 2016, 2017) and in adolescents with ADHD (Stevens et al., 2016). Three meta-analyses have been conducted investigating near-transfer effects of CWMT on working memory (Shipstead et al., 2012; Melby-Lervåg and Hulme, 2013; Aksayli et al., 2019). Two out of these three meta-analyses concluded that there is evidence that CWMT leads to improved working memory task performance (Melby-Lervåg and Hulme, 2013; Aksayli et al., 2019), with the strength of the improvement depending on the similarity of the tasks to the training tasks (Aksayli et al., 2019). Four meta-analyses have been conducted investigating far-transfer effects of CWMT on untrained functions (Shipstead et al., 2012; Melby-Lervåg and Hulme, 2013; Spencer-Smith and Klingberg, 2016; Aksayli et al., 2019). Of these, three meta-analyses concluded that there is no evidence for improvements of untrained functions after following CWMT (Shipstead et al., 2012; Melby-Lervåg and Hulme, 2013; Aksayli et al., 2019). Only one randomized controlled trial into effects of CWMT in very preterm born children has been conducted and showed no improvements in academic achievement, working memory, attention, daily life EF and general cognitive ability (Anderson et al., 2018). However, CWMT is an EF training program that focuses solely on training working memory, while other core EFs such as inhibition and cognitive flexibility are also affected in children born preterm (Aarnoudse-Moens et al., 2009; Mulder et al., 2009; van Houdt et al., 2019).

Recently, a game-formatted and comprehensive EF training program entitled BrainGame Brian (BGB) was developed, that aimed at training not only working memory, but also inhibition and cognitive flexibility, in children aged 8–12 years (Prins et al., 2013). BrainGame Brian involves a game-world in which training tasks for visuospatial working memory, response inhibition and cognitive flexibility are played to help the main character, Brian. Difficulty level is automatically adjusted according to the child’s performance. The training program is played four times a week for 30–45 min per session. The BGB EF training program has been consistently shown to improve working memory in children with ADHD and Autism Spectrum Disorder (ASD) (van der Oord et al., 2014; de Vries et al., 2015; Dovis et al., 2015). However, effects on other EFs or other untrained functions were inconsistent (van der Oord et al., 2014; de Vries et al., 2015; Dovis et al., 2015). Furthermore, one small-sized non-randomized pilot study has been conducted into the feasibility of the BGB EF training program in very preterm children, which showed positive effects on visuospatial working memory task performance (Aarnoudse-Moens et al., 2018). The BGB EF training program may have beneficial effects on various areas of functioning, including attentional, behavioral and emotional functioning and self-perceived competence in very preterm born children. Deficits in EF have been shown to play a crucial role in a range of psychiatric disorders such as ADHD and ASD, and a large body of literature has indicated that executive functioning is strongly related to both behavioral and emotional functioning (Ozonoff et al., 1991; Pennington and Ozonoff, 1996; Nigg, 2000; Sergeant et al., 2002; Oosterlaan et al., 2005; Willcutt et al., 2005; Riggs et al., 2006; Carlson and Wang, 2007). In very preterm children, deficits in EF have been shown to underlie the attentional problems these children encounter as well (Mulder et al., 2011; de Kieviet et al., 2012; Aarnoudse-Moens et al., 2013). Therefore, improving EFs with the BGB EF training program could lead to improvements in attentional, behavioral and emotional functioning as well. If the BGB EF training program leads to improvement in those domains, it may improve children’s self-perceived competence as well.

Therefore, the current study aimed to investigate effects of the BGB EF training program on attentional functioning, parent and teacher rated behavioral and emotional functioning and self-perceived competence in a group of very preterm (<30 weeks of gestation) and/or extremely low birthweight (< 1000 g) children with parent-rated attention problems, compared to both a placebo training and waitlist arm. The BGB EF training program uses game elements and strong and immediate reinforcements to optimize the participants’ motivational state and compliance with the training, which in turn is supposed to enhance efficacy of the training. The effects of EF training with BGB may therefore be moderated by exposure to gaming before start of the EF training program. More specifically, children with intensive exposure to gaming may show a more blunted response to the reinforcements build in the training than children with little exposure to gaming. Therefore, exploratory analyses also examined effects of the BGB EF training program while correcting for time spent gaming outside school-hours. Also, associations between time spent gaming outside school-hours and baseline measurements were examined.

## Materials and Methods

### Trial Design

This was a multi-center, double-blind, placebo and waitlist-controlled randomized trial conducted in two academic hospitals in The Netherlands (Amsterdam University Medical Centers and University Medical Center Utrecht). The Medical Ethical Committee of the two participating academic hospitals approved the study protocol and the execution of the study procedures was according to the Declaration of Helsinki. The trial was registered in the Dutch Trial Registry (NTR, # NTR5365). CONSORT guidelines were followed.

### Participants

**FIGURE 1:**
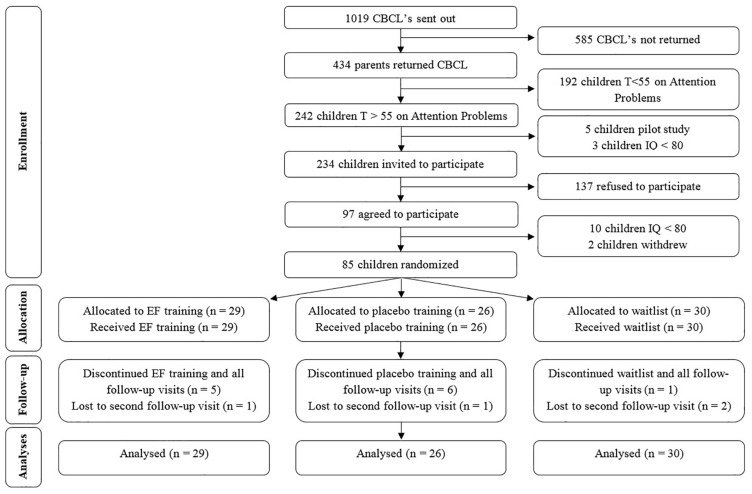
CONSORT Flow diagram. CBCL, Child Behavior CheckList; IQ, Intelligence Quotient. EF, Executive Function.

The Dutch version of the Child Behavior Checklist 6–18 years (CBCL6-18) (Verhulst and Van der Ende, 2013) was sent to parents of 7–12 year old (chronological age) children born very preterm (<30 weeks of gestation) and/or with extremely low birthweight (birthweight < 1000 g) that participated in the national neonatal follow-up program after being admitted to the Neonatal Intensive Care Unit (NICU) in one of the two participating hospitals. Eligible for this study were children of whom parents reported attention problems on the CBCL6-18 (T ≥ 55 on the Attention Problems scale, Hudziak et al., 2004), as soon as they reached the chronological age of at least 8 years. Exclusion criteria were an estimated IQ < 80 (in order to assure that the child was able to understand and comply with instructions), motor problems too profound to allow use of a computer and no Dutch language use in the home situation. The inclusion process and participant’s flow through the study is depicted in Figure 1. Reasons not to return the questionnaire that was used to assess whether children had parent- rated attention problems were no time or no interest. Reasons not to participate were that parents found that incorporation of the training sessions into already busy schedules was too burdensome for the child and/or family or that parents or children had no interest in participating. In short, 85 children were randomized, 29 to the EF training arm, 26 to the placebo training arm and 30 to the waitlist arm. Data of the first follow-up visit were available for 24, 20, and 29 children, respectively, and data of the second follow-up visit were available for 23, 19, and 27 children, respectively. Thus of all children, 81% completed all assessments. Reasons for withdrawal from the study after randomization were not being able to incorporate training sessions into a busy schedule or the child not wanting to complete the training sessions (*n* = 9), no time or willingness to schedule the follow-up visit(s) at the appropriate time-point(s) (*n* = 5) or severe illness discovered (*n* = 2). All available data of participants (also data of participants with missing data) were incorporated in the analyses.

### Randomization and Blinding

Children meeting inclusion criteria were randomly assigned to one of three treatment arms: EF training, placebo training or waitlist. Allocation to treatment arms was stratified by age (below or above 10.5 years of age) and severity of attention problems (Attention Problems T-score below or above 65), with equal proportions of children allocated to each arm within the same stratum. A random number generator was used to generate randomization lists. A researcher not otherwise involved in this study was responsible for randomization and handed the test assistant a sealed envelope with a note stating ‘waitlist’ or a login and password, which was opened by the child and parents after baseline assessment. To ensure blinding, parents were only informed about whether their child was randomized to either one of two training arms or the waitlist arm, and in case more children from the same family were included in the study, one of those was randomized and the other was put in the same arm. All staff was blinded to EF training or placebo training assignment, including the person involved in randomization. Test assistants that played the first training session with the child were deblinded because of differences in training tasks (see below) between EF training and placebo training and were not involved in follow-up assessments of these children. Parents, children and researchers were aware of children’s allocation to the waitlist arm. Data were analyzed by a researcher blinded to treatment allocations.

### Intervention

#### BrainGame Brian Training

The BGB EF training program is a game-formatted, computerized training program (Prins et al., 2013) that is performed by the child at home. The BGB EF training program uses game elements and strong and immediate reinforcements to optimize the participants’ motivational state and compliance with the training. The game-world exists of several different villages, in each of which there are characters that face problems and need help of the main character: Brian. During the first sessions, only one of those villages in accessible, with more villages becoming accessible during the course of the training program. To help the characters facing problems, children perform the EF training tasks with Brian. After completion of each block of training tasks, an invention made by Brian will appear in the game-world that helps solving the problem of the character, thereby acting as an immediate reinforcement. These inventions remain visible in the game-world during subsequent sessions. Thus the more sessions children have performed, the more inventions will be visible in the game-world, which enhances motivation. The training consists of 25 sessions, with two blocks of three training tasks, one for each EF, administered in every session. These three training tasks remain the same throughout the 25 sessions, except for the visuospatial working memory task, which was administered in five different versions to increase working memory demands.

#### EF Training Arm

In the working memory task, children are asked to repeat a sequence of dots on a 4 × 4 grid. There were five versions of the working memory task, each of which was administered for five consecutive training sessions and increased in difficulty level across training sessions. In the inhibition task, children are asked to press a button in a specific time window (target), but to refrain from pressing that button when a visual stop signal is presented. In the cognitive flexibility task, children are asked to sort objects according to either it’s shape or it’s color, with the sorting rule changing every three to five trials. Difficulty level of each training task is automatically adjusted to the child’s level of performance. The number of trials and therefore also the duration of all three tasks depends on the child’s performance. Most children are able to finish the training tasks within 8 min per task. For the first three and last two versions of the working memory task, a total of at least 74 and 62 boxes need to be repeated correctly (with only correctly repeated boxed within correctly repeated sequences adding to this total), respectively, to end the task. For the inhibition task, the task ends after ten blocks of five trials that were all performed correctly. For the cognitive flexibility task, the task ends after 10 blocks of three-to-five trials that were all performed correctly. Difficulty level was adjusted for each task after completion. Difficulty level for the working memory task was adjusted by increasing or decreasing the sequence length. Difficulty level for the inhibition task was adjusted by increasing or decreasing the time between start of the time window in which children needed to respond and presentation of the stop signal. Difficulty for the cognitive flexibility task was adjusted by increasing or decreasing the time children have to sort each presented target.

#### Placebo Training Arm

The placebo training arm is identical to the actual training arm, however, the specific elements that actually train the EFs are removed from the training tasks. In the working memory task, children are asked to repeat sequences with a span length of two in the same order as presented. In that way, the training task only involves short-term memory and does not tax working memory. In the inhibition task, no stop-signals are presented. In the shifting task, no shifting trials are presented. Furthermore, difficulty level is not adjusted. Thus, children do play the training tasks, but do not train working memory, inhibition or cognitive flexibility in the placebo training arm.

#### Waitlist Arm

Children in the waitlist arm do not play the training and were instructed to perform the same activities in the waiting period as they do normally.

### Measures

#### Improvement During Training

To validate whether the BGB EF training program actually induced improvement on the trained tasks, we assessed improvement of training performance across all training sessions. For the inhibition and cognitive flexibility tasks, improvement was assessed by comparing the mean difficulty level of day two and day three of training (start level) with the highest achieved difficulty level (highest level). All children start at the same level at day one, but for some children this level is too easy and for some children this level is too difficult. Therefore, taking the mean difficulty level of day two and day three as start level ensures that this is the child’s actual level of performance at the beginning of the training. As there were five versions of the working memory task, which were each played in five consecutive training sessions, for each version improvement was assessed by comparing mean difficulty level at day two (start level) with the highest achieved difficulty level (highest level). Again, mean difficulty level at day two was chosen as start level to ensure this was the child’s actual level of performance at the start of each new version of the working memory task. Difficulty level at day two and not mean difficulty level of day two and three (as was done for the inhibition and cognitive flexibility tasks) was chosen because each version of the working memory task was only performed in five consecutive training sessions and not 25 as in the inhibition and cognitive flexibility tasks.

#### Attentional Functioning

The Child version of the Attention Network Test (Child-ANT) (Rueda et al., 2004) was administered to assess efficiency of the three attention networks: (1) the alerting network, (2) the orienting network, and (3) the executive attention network. Each trial of the Child-ANT started with a central fixation cross. The target was one single yellow fish or a horizontally positioned line of five yellow fish, appearing above or below the fixation cross. The child was asked to respond by pressing one of two buttons on the side the central fish pointed to. Trials could be (a) congruent (central fish pointing to same direction as flanking fish), (b) incongruent (central fish pointing to opposite direction as flanking fish) or (c) neutral (only central fish, no flanking fish). Furthermore, each target was preceded by a warning cue condition that comprised one of four options: (a) no cue, (b) center cue (cue presented at the location of the fixation cross), (c) double cue (cues presented above and below the fixation cross), or (d) spatial cue (cue presented at the location of the upcoming target). Outcome measures were efficiency of the alerting, orienting and executive attention networks, calculated by (1) subtracting the median RT for the double cue condition from the median RT for the no cue condition, (2) subtracting the median RT for the spatial cue from the median RT for the central cue and (3) subtracting the median RT for the congruent trials from the median RT for the incongruent trials, respectively. For the alerting and orienting networks, higher values reflect higher network efficiency. For the executive attention network, higher values reflect lower network efficiency.

#### Behavioral and Emotional Functioning

Behavioral and emotional functioning was measured with the Strengths and Difficulties Questionnaire (SDQ) (van Widenfelt et al., 2003) which contains five subscales: Emotional Problems, Hyperactivity, Conduct Problems, Peer Problems and Prosocial Behavior. Raw scores on these scales were used as outcome measures. Scores may range between 0 and 10, with higher scores reflecting more problems.

#### Self-Perceived Competence

The Dutch translation of the Self-Perception Profile for Children (CBSK) (Veerman et al., 1997), was used to assess self-perceived competence using six scales: Scholastics, Social Acceptance, Athletics, Physical Appearance, Behavioral Conduct and Global Self-Worth. Raw scores on these scales were used as outcome measures. Scores may range between 6 and 24, with higher scores reflecting higher self-perceived competence.

#### Gaming at Baseline

Gaming was defined as playing games on any electronic apparatus. At the baseline assessment, parents provided information on the amount of hours per week their children spent gaming outside of school-hours.

### Procedure

After written informed consent was obtained from parents and, if applicable, from children aged 12, children completed a baseline neurocognitive assessment including estimated IQ, efficiency of attention networks and self-perceived competence. Parents and teachers were asked to fill out a questionnaire on children’s behavioral and emotional functioning (a full description is provided below). Assessments were part of a larger battery of measures administered to study effectiveness of the BGB EF training program. When children were randomized to either the EF training or placebo training arm, a house visit was made to install the BGB EF training program at the home computer or laptop and play the first session. When children were randomized to the waitlist-control arm, no house visit was made. To assess short-term and longer-term efficacy of the BGB EF training program, two follow-up visits were scheduled. The first follow-up assessment (T1) was approximately 2 weeks after the last training session (approximately 2 months after baseline assessment for children in the waitlist condition) and the second follow-up assessment (T2) was approximately 5 months after the first follow-up assessment. Gaming at baseline, demographic characteristics, medical characteristics of the neonatal period and estimated IQ were only assessed at baseline assessment. Attentional functioning, behavioral and emotional functioning and self-perceived competence were assessed at baseline assessment and both follow-up assessments.

### Demographic Characteristics, Medical Characteristics of Neonatal Period and IQ

Parents provided information on demographics. Medical data from the neonatal period were obtained from medical records. To estimate IQ, a two subtest short-form (Vocabulary and Block Design) of the Dutch Wechsler Intelligence Scale for Children, Third Edition (WISC-III-NL, Sattler, 1992), was administered during the baseline assessment. Scaled scores for both the Vocabulary and Block Design subtests were computed. Subsequently the estimated full scale IQ equivalent for the sum of scaled scores of these two subtests was taken from the manual. Estimated IQ based on this short-form correlates highly with full scale IQ (*r* = 0.90) (Sattler, 1992).

### Statistical Analyses

Sample size calculation was based on a repeated measures design with three time points. To be able to demonstrate a medium-sized intervention effect (Cohen’s *d* = 0.5), assuming a within-subject correlation of 0.295 (taken from our BGB EF training pilot study in very preterm, Aarnoudse-Moens et al., 2018), a power of 80% and a significance level of 0.05, 39 children in each intervention arm were needed (Twisk, 2013).

IBM SPSS Statistics version 25 was used for the statistical analyses (IBM, 2017). Outliers were winsorized at three standard deviations (SDs) (Ghosh and Vogt, 2012). For baseline assessment, first follow-up assessment and second follow-up assessment, 4.7, 11, and 10.1% of data was missing for the Child-ANT, respectively, 1.2, 1.4, and 5.8% of data was missing for the parent SDQ, respectively, 14.1, 44.7, and 52.2% of data was missing for the teacher SDQ respectively, and 3.5, 2.7, and 1.4% of data was missing for the CBSK, respectively. Missing data were not imputed.

Data were analyzed on intention-to-treat basis. To assess whether attrition from the study was selective, children that did and did not complete all assessments were compared on all demographic and neonatal medical characteristics and all outcome measures with independent *t*-tests and chi-square tests. To assess whether demographic and neonatal medical baseline characteristics differ between the treatment arms, one-way analyses of variance (ANOVA’s) and chi-square tests were performed. To assess whether children actually improved on the training tasks in the BGB EF training program, their start level was compared to their highest level for the inhibition task, cognitive flexibility task and each of five versions of the working memory task with paired *t*-tests.

To assess whether there was a differential effect of treatment arm over time on attentional functioning, behavioral and emotional functioning, and self-perceived competence, linear mixed model analyses were run for all outcome measures with a random intercept to account for dependency in the data due to family bonds, and fixed factors for treatment arm, time and the interaction between treatment arm and time. To assess whether differential effects of treatment arm over time existed for younger and older children, linear mixed model analyses were performed on all outcome measures. A random intercept accounted for dependency in the data due to family bonds and the three-way interaction between treatment arm, time and age above or below 10.5 years was added as a fixed factor. To assess whether effects of BGB EF training program depend on time spent gaming before start of the training, the described linear mixed model analyses were also run with time spent gaming outside school-hours at baseline assessment added as a covariate. All available data was used in all linear mixed model analyses. In addition, we explored the association between gaming at baseline and baseline measurements of attentional, behavioral and emotional functioning and self-perceived competence, using Pearson *r* correlations.

## Results

### Preliminary Analyses

Attrition analyses showed no differences on any of the demographic or neonatal medical characteristics nor on any of the outcome measures at baseline between children that did and did not complete all assessments (all *t*-values < 1.94, all χ*^2^*-values < 0.72, all *p*-values > 0.06). There were no significant differences on any of the baseline demographics or neonatal medical characteristics between the treatment arms, with one single exception. There was a significant difference between the treatment arms for open ductus botalli that was treated with either medication or surgery [χ*^2^*(2) = 10.2, *p* = 0.006], with less children with a treated open ductus botalli in the EF training arm than in the placebo and waitlist arm. An open ductus botalli is very common in preterm neonates, with an incidence of 50% in infants born with a birthweight below 750 g and 37% in infants born with a birthweight between 750 and 1000 g (Dice and Bhatia, 2007). Treated open ductus botalli has been found to be not associated with neurodevelopmental outcomes (Chorne et al., 2007). See Table 1 for more detailed information on the demographic and neonatal characteristics of the three treatment groups at baseline. Assessments took place between October 2015 (first baseline measurement) and September 2018 (last second follow-up measurement). Mean number of weeks between baseline assessment and first follow-up assessment was 9.1 weeks (*SD* = 2.5) and mean number of weeks between baseline assessment and second follow-up assessment was 32.7 weeks (*SD* = 4.8). Mean number of months between first and second follow-up assessment was 5.5 months (*SD* = 0.8 months). There were no significant differences in time between baseline and first or second follow-up assessments between the three treatment arms [*F*(2,68) = 0.66, *p* = 0.52; *F*(2,60) = 2.0, *p* = 0.15, respectively].

**TABLE 1:** Demographic and neonatal medical characteristics for the three treatment groups.

**Measure**	**EF training (*n* = 29)**	**Placebo training (*n* = 26)**	**Waitlist (*n* = 30)**	**Test statistic (df), *p*-value**
**Demographic characteristics**				
GA (M, SD)	28.2 (1.3)	28.0 (1.0)	27.8 (1.4)	*F*(2,82) = 0.67, *p* = 0.52
BW (M, SD)	1026 (256)	1039 (179)	1049 (267)	*F*(2,82) = 0.07. *p* = 0.93
Age (M, SD)	10.2 (1.2)	10.2 (1.3)	10.3 (1.1)	*F*(2,82) = 0.03, *p* = 0.97
IQ (M, SD)	99.0 (13.6)	96.4 (11.7)	100.8 (11.1)	*F*(2,82) = 0.95, *p* = 0.39
CBCL attention T-score (M, SD)	62.8 (6.9)	64.0 (7.6)	64.4 (7.0)	*F*(2,82) = 0.38, *p* = 0.69
Time spent gaming	5.5 (5.9)	8.0 (6.3)	8.5 (7.0)	*F*(2,82) = 1.7, *p* = 0.18
Boys (n,%)	13(44%)	16(62%)	20(67%)	χ*^2^*(2) = 3.1, *p* = 0.21
Parental education level (n,%)				χ*^2^*(4) = 8.9, *p* = 0.06
Low	6(21%)	4(16%)	1(4%)	
Middle	3(10%)	5(20%)	11(39%)	
High	20(69%)	16(54%)	16(57%)	
**Neonatal medical characteristics**				
SGA (n,%)	8(28%)	4(17%)	4(14%)	χ*^2^*(2) = 1.9, *p* = 0.38
Ventilator support (n,%)	20(69%)	17(65%)	23(77%)	χ*^2^*(2) = 0.9, *p* = 0.64
BPD at 36 weeks PMA (n,%)	7(24%)	4(16%)	6(21%)	χ*^2^*(2) = 0.2, *p* = 0.90
IVH I or II	9(31%)	6(23%)	8(27%)	χ*^2^*(2) = 0.4, *p* = 0.80
IVH III or IV	0(0%)	2(8%)	1(3%)	χ*^2^*(2) = 2.4, *p* = 0.30
PVL I	1(3%)	2(8%)	0(0%)	χ*^2^*(2) = 2.4, *p* = 0.30
PVL II, III or IV	0(0%)	0(0%)	0(0%)	
Open Ductus Botalli treated	3(10%)	12(46%)	13(43%)	χ*^2^* (2) = 10.2, *p* < 0.01
Sepsis	17(59%)	16(62%)	20(67%)	χ*^2^*(2) = 0.4, *p* = 0.81

*GA, Gestational Age; BW, Birth Weight; IQ, Intelligence Quotient; CBCL, Child Behavior CheckList; SGA, Small for Gestational Age; BPD, BronchoPulmonary Dysplasia; PMA, Post Menstrual Age; IVH, IntraVentricular Hemorrhage; PVL, PeriVentricular Leukomalacia; M, mean; SD, standard deviation; *n*, number.*

### Improvement During Training

For the inhibition training task, the cognitive flexibility training task and all five versions of the working memory task, significant improvements were found across the training sessions in the EF training arm. Performance significantly increased on all measures between the start level and the highest level achieved of children, indicating that children actually improved on all training tasks over the course of the EF training program. See Table 2 for more details.

**TABLE 2:** Improvement during training on the training tasks.

**Training task**	**Start level**	**Highest level**	**Test statistic,**
	***M* (*SD*)**	***M* (*SD*)**	***p*-value**
Working memory version 1	3.15 (0.42)	3.87 (0.65)	*t*(23) = −8.97, *p* < 0.001^∗^
Working memory version 2	3.25 (0.37)	3.84 (0.71)	*t*(23) = −6.87, *p* < 0.001^∗^
Working memory version 3	3.07 (0.41)	4.03 (0.82)	*t*(23) = −8.75, *p* < 0.001^∗^
Working memory version 4	2.95 (0.44)	3.55 (0.77)	*t*(23) = −7.56, *p* < 0.001^∗^
Working memory version 5	2.98 (0.53)	3.74 (0.96)	*t*(22) = −6.89, *p* < 0.001^∗^
Inhibition	3.70 (1.30)	11.42 (2.08)	*t*(23) = −18.39, *p* < 0.001^∗^
Cognitive Flexibility	1.28 (0.51)	8.63 (4.14)	*t*(23) = −8.44, *p* < 0.001^∗^

*^∗^Significant at α = 0.05. M, mean; SD, standard deviation.*

### Effects of the EF Training Program on Attentional, Behavioral and Emotional Functioning and Self-Perceived Competence

There was no significant difference over time between the three treatment arms for efficiency of the orienting and executive attention networks. The difference over time between the three treatment arms for the alerting network approached significance [*F*(4,133) = 2.40, *p* = 0.053]. *Post hoc* mixed model analyses indicated larger improvement of alerting network efficiency in the waitlist arm than in the EF training arm between baseline and first follow-up assessment, but larger improvement in EF training arm than in the waitlist arm between first and second follow-up assessment. There were significant main effects of time for efficiency of the executive network [*F*(2,139) = 9.34, *p* < 0.001] and the alerting network [*F*(2,133) = 7.51, *p* = 0.001], indicating efficiency improved over time. See Table 3.

**TABLE 3:** Baseline and follow-up data on the Attention Network Test for Children for the three treatment groups.

**Outcome measure**	**T0 *M* (*SE*; 95% CI) *N* = 85**	**T1 *M* (*SE*; 95% CI) *N* = 73**	**T2 *M* (*SE*; 95% CI) *N* = 69**	***p*-value**
**Attention Network Test**				
**Orienting Network**				
EF training	26.15 (9.29; 7.83 – 44.47)	22.29 (10.68; 1.22 – 43.36)	21.08 (11.23; −1.06 – 43.22)	Group: 0.18
Placebo training	52.45 (9.77; 33.18 – 71.72)	38.54 (12.58; 13.72 – 3.36)	22.79 (11.83; −0.54 – 46.11)	Time: 0.22
Waitlist	25.19 (9.25; 6.94 – 43.44)	21.07 (9.10; 3.14 – 39.01)	18.42 (9.60; −0.51 – 37.36)	Group × Time: 0.77
**Alerting Network**				
EF training	74.57 (8.63; 57.54 – 91.60)	50.22 (9.86; 30.78 – 69.66)	90.90 (10.34; 70.51 – 111.28)	Group:0.58
Placebo training	53.46 (9.03; 35.64 – 71.28)	64.45 (11.54; 41.70 – 87.20)	79.18 (10.87; 57.74 – 100.61)	Time: 0.001
Waitlist	55.35 (8.56; 38.47 – 72.23)	79.45 (8.42; 62.84 – 96.07)	90.70 (8.87; 73.21 – 108.18)	Group × Time: 0.053
**Executive Network**				
EF training	85.90 (9.60; 66.95 – 104.83)	57.12 (10.98; 35.47 – 78.77)	58.04 (11.52; 35.32 – 80.76)	Group:0.25
Placebo training	97.76 (10.05; 77.93 – 117.59)	65.69 (12.86; 40.33 – 91.06)	61.26 (12.11; 37.37 – 85.15)	Time: <0.001^∗^
Waitlist	73.86 (9.53; 55.07 – 92.65)	45.58 (9.37; 27.09 – 64.07)	55.38 (9.87; 35.91 – 74.85)	Group × Time: 0.91

*^∗^Significant at α = 0.05. Depicted are estimated marginal Means (M) and Standard Errors (SE). CI, Confidence Interval; N, total number of participants; T0, Time-point 0, i.e., baseline; T1, Time-point 1, i.e., first follow-up visit; T2, Time-point 2, i.e., second follow-up visit. See Figure 1 for number of participants in each group at each time-point.*

There was no significant difference over time between the three treatment arms for any of the subscales of parent or teacher Strengths and Difficulties questionnaire. There was a significant main effect of treatment arm for the teacher Peer Problems subscale, indicating less peer problems in the EF training arm than in the waitlist arm [*F*(2,77) = 3.65, *p* = 0.03]. See Table 4.

**TABLE 4:** Baseline and follow-up data on the Strengths and Difficulties Questionnaire according to parents and teachers for the three treatment groups.

**Outcome measure**	**T0 *M* (*SE*; 95% CI) *N* = 85**	**T1 *M* (*SE*; 95% CI) *N* = 73**	**T2 *M* (*SE*; 95% CI) *N* = 69**	***p*-values**
**Parent SDQ**				
**Emotional Symptoms**				
EF training	2.79 (0.45; 1.90 – 3.68)	2.35 (0.47; 1.42 – 3.28)	2.67 (0.48; 1.72 – 3.61)	Group: 0.19
Placebo training	3.46 (0.45; 2.56 – 4.36)	2.70 (0.49; 1.72 – 3.67)	2.90 (0.51; 1.90 – 3.90)	Time: 0.07
Waitlist	2.23 (0.42; 1.39 – 3.06)	1.92 (0.43; 1.07 – 2.77)	1.86 (0.44; 0.98 – 2.73)	Group × Time:0.88
**Conduct Problems**				
EF training	1.03 (0.36; 0.31 – 1.75)	0.72 (0.38; −0.02 – 1.47)	0.93 (0.38; 0.18 – 1.69)	Group: 0.13
Placebo training	1.68 (0.37; 0.95 – 2.40)	1.80 (0.39; 1.03 – 2.58)	1.84 (0.41; 1.03 – 2.65)	Time: 0.92
Waitlist	1.55 (0.34; 0.87 – 2.23)	1.81 (0.35; 1.12 – 2.50)	1.69 (0.35; 0.99 – 2.39)	Group × Time:0.68
**Peer Problems**				
EF training	1.51 (0.39; 0.74 – 2.28)	1.24 (0.40; 0.44 – 2.03)	1.05 (0.41; 0.25 – 1.86)	Group: 0.18
Placebo training	2.07 (0.39; 1.29 – 2.84)	1.50 (0.42; 0.67 – 0.33)	1.97 (0.44; 1.11 – 2.83)	Time: 0.13
Waitlist	2.33 (0.36; 1.61 – 3.05)	2.24 (0.37; 1.51 – 2.98)	1.93 (0.38; 1.19 – 2.67)	Group × Time: 0.50
**Prosocial Behavior**				
EF training	8.76 (0.36; 8.04 – 9.48)	8.41 (0.38; 7.66 – 9.16)	8.54 (0.39; 7.77 – 9.30)	Group: 0.35
Placebo training	8.20 (0.37; 7.47 – 8.93)	7.85 (0.40; 7.06 – 8.65)	7.68 (0.42; 6.86 – 8.50)	Time: 0.32
Waitlist	8.14 (0.34; 7.46 – 8.81)	8.01 (0.35; 7.32 – 8.70)	8.19 (0.36; 7.49 – 8.90)	Group × Time: 0.83
**Hyperactivity**				
EF training	5.26 (0.45; 4.37 – 6.15)	4.76 (0.47; 3.83 – 5.69)	4.67 (0.48; 3.71 – 5.62)	Group: 0.04^∗^
Placebo training	6.44 (0.46; 5.53 – 7.35)	6.37 (0.50; 5.38 – 7.36)	5.56 (0.52; 4.54 – 6.58)	Time: 0.10
Waitlist	6.24 (0.42; 5.40 – 7.07)	6.15 (0.43; 5.29 – 7.00)	6.01 (0.44; 5.14 – 6.89)	Group × Time: 0.73
**Teacher SDQ**				
**Emotional Symptoms**				
EF training	1.90 (0.40; 1.10 – 2.70)	1.66 (0.43; 0.80 – 2.52)	1.73 (0.45; 0.84 – 2.62)	Group: 0.62
Placebo training	1.51 (0.43; 0.65 – 2.37)	1.58 (0.49; 0.61 – 2.55)	1.59 (0.61; 0.40 – 2.79)	Time: 0.83
Waitlist	1.38 (0.39; 0.60 – 2.16)	1.23 (0.43; 0.38 – 2.08)	1.12 (0.47; 0.20 – 2.04)	Group × Time: 0.97
**Conduct Problems**				
EF training	0.66 (0.27; 0.13 – 1.18)	0.41 (0.30; −0.18 – 1.01)	0.40 (0.32; −0.23 – 1.03)	Group: 0.53
Placebo training	0.76 (0.30; 0.17 – 1.35)	0.99 (0.35; 0.29 – 1.69)	0.75 (0.47; −0.18 – 1.67)	Time: 0.56
Waitlist	1.01 (0.26; 0.50 – 1.52)	0.89 (0.29; 0.31 – 1.47)	0.59 (0.34; −0.09 – 1.26)	Group × Time:0.82
**Peer Problems**				
EF training	1.15 (0.39; 0.38 – 1.92)	0.61 (0.43; −0.25 – 1.47)	0.85 (0.47; −0.08 – 1.78)	Group: 0.03^∗^
Placebo training	2.05 (0.43; 1.20 – 2.91)	1.97 (0.51; 0.97 – 2.98)	1.54 (0.67; 0.22 – 2.85)	Time: 0.24
Waitlist	2.43 (0.38; 1.69 – 3.18)	1.97 (0.43; 1.13 – 2.81)	2.11 (0.49; 1.14 – 3.07)	Group × Time: 0.93
**Prosocial Behavior**				
EF training	7.94 (0.48; 6.99 – 8.89)	8.34 (0.52; 7.31 – 9.37)	7.57 (0.55; 6.49 – 8.65)	Group: 0.42
Placebo training	7.83 (0.51; 6.81 – 8.85)	7.13 (0.60; 5.95 – 8.31)	7.46 (0.76; 5.97 – 8.96)	Time: 0.23
Waitlist	7.58 (0.46; 6.66 – 8.50)	6.93 (0.51; 5.92 – 7.93)	6.85 (0.57; 5.73 – 7.98)	Group × Time: 0.35
**Hyperactivity**				
EF training	4.39 (0.60; 3.21 – 5.57)	3.90 (0.65; 2.60 – 5.19)	4.14 (0.69; 2.78 – 5.50)	Group: 0.70
Placebo training	5.51 (0.66; 4.20 – 6.82)	5.22 (0.76; 3.72 – 6.73)	2.64 (0.97; 0.72 – 4.56)	Time: 0.02^∗^
Waitlist	5.09 (0.58; 3.94 – 6.23)	4.71 (0.64; 3.44 – 5.98)	4.61 (0.74; 3.15 – 6.08)	Group × Time: 0.14

*^∗^Significant at α = 0.05. Depicted are estimated marginal means (M) and standard errors (SD). CI, Confidence Interval; N, total number of participants; SDQ, Strengths and Difficulties Questionnaire; T0, Time-point 0, i.e., baseline; T1, Time-point 1, i.e., first follow-up visit; T2, Time-point 2, i.e., second follow-up visit. See Figure 1 for number of participants in each group at each time-point.*

There was no significant difference over time between the three treatment arms and time for any of the subscales of the self-perceived competence questionnaire for children. There were significant main effects of time for self-perceived competence in Scholastics [*F*(2,144) = 6.04, *p* = 0.003] and Athletic Competence [*F*(2,142) = 3.42, *p* = 0.04], both suggesting improved self-perceived competence over time. The main effect of time for self-perceived Behavioral Conduct approached significance [*F*(2,145) = 2.95, *p* = 0.06], suggesting improved self-perceived competence over time. See Table 5.

**TABLE 5:** Baseline and follow-up data on self-perceived competence for the three treatment groups.

**Domain**	**T0 *M* (*SE*; 95% CI) *N* = 85**	**T1 *M* (*SE*; 95% CI) *N* = 73**	**T2 *M* (*SE*; 95% CI) *N* = 69**	***p*-value**
**Scholastics**				
EF training	15.12 (0.70; 13.74 – 16.50)	16.01 (0.74; 14.55 – 17.47)	14.56 (0.75; 13.08 – 16.04)	Group: 0.52
Placebo training	15.37 (0.73; 13.93 – 16.81)	17.15 (0.79; 15.59 – 18.72)	15.72 (0.79; 14.16 – 17.29)	Time: 0.003^∗^
Waitlist	15.50 (0.66; 14.21 – 16.80)	16.59 (0.66; 15.28 – 17.90)	16.18 (0.68; 14.84 – 17.51)	Group × Time: 0.58
**Social Acceptance**				
EF training	18.54 (0.85; 16.86 – 20.21)	18.18 (0.89; 16.43 – 19.93)	17.79 (0.90; 16.02 – 19.57)	Group: 0.08
Placebo training	16.68 (0.88; 14.94 – 18.41)	17.30 (0.94; 15.45 – 19.16)	16.29 (0.94; 14.43 – 18.15)	Time: 0.31
Waitlist	19.19 (0.79; 17.62 – 20.75)	19.43 (0.80; 17.85 – 21.01)	18.95 (0.81; 17.34 – 20.56)	Group × Time: 0.90
**Athletic Competence**				
EF training	17.34 (0.71; 15.93 – 18.75)	17.35 (0.75; 15.86 – 18.84)	17.98 (0.76; 16.47 – 19.49)	Group: 0.38
Placebo training	17.63 (0.74; 16.16 – 19.10)	18.04 (0.80; 16.45 – 19.63)	18.50 (0.80; 16.92 – 20.09)	Time: 0.04^∗^
Waitlist	17.68 (0.67; 16.35 – 19.00)	19.55 (0.68; 18.21 – 20.89)	19.02 (0.69; 17.66 – 20.39)	Group × Time: 0.29
**Physical Appearance**				
EF training	19.19 (0.82; 17.56 – 20.81)	18.89 (0.85; 17.20 – 20.58)	18.82 (0.86; 17.12 – 20.53)	Group: 0.07
Placebo training	19.54 (0.85; 17.86 – 21.22)	19.32 (0.90; 17.54 – 21.10)	19.44 (0.90; 17.66 – 21.22)	Time: 0.83
Waitlist	20.56 (0.77; 19.03 – 22.08)	21.53 (0.77; 20.00 – 23.07)	21.65 (0.78; 20.09 – 23.20)	Group × Time: 0.36
**Behavioral Conduct**				
EF training	17.63 (0.72; 16.20 – 19.06)	18.34 (0.77; 16.82 – 19.85)	17.65 (0.78; 16.11 – 19.19)	Group: 0.78
Placebo training	17.26 (0.76; 15.76 – 18.75)	18.46 (0.82; 16.83 – 20.09)	17.42 (0.82; 15.79 – 19.05)	Time: 0.06
Waitlist	18.06 (0.68; 16.71 – 19.40)	18.89 (0.69; 17.53 – 20.25)	17.94 (0.70; 16.55 – 19.33)	Group × Time: 0.99
**Global Self-Worth**				
EF training	20.07 (0.65; 18.78 – 21.37)	19.97 (0.69; 18.61 – 21.33)	20.60 (0.70; 19.23 – 21.98)	Group: 0.12
Placebo training	19.97 (0.68; 18.62 – 21.32)	20.55 (0.73; 19.11 – 22.00)	20.60 (0.73; 19.16 – 22.05)	Time: 0.27
Waitlist	21.35 (0.61; 20.14 – 22.57)	22.11 (0.62; 20.89 – 23.34)	21.69 (0.63; 20.45 – 22.94)	Group × Time: 0.70

*^∗^Significant at α = 0.05. Depicted are estimated marginal means (M) and standard errors (SD). CI, Confidence Interval; N, total number of participants; T0, Time-point 0, i.e., baseline; T1, Time-point 1, i.e., first follow-up visit; T2, Time-point 2, i.e., second follow-up visit. See Figure 1 for number of participants in each group at each time-point.*

Significant three-way interactions between treatment arm, time, and age (above or below 10.5 years) were found for the alerting and executive attention networks [*F*(17,121) = 1.89, *p* = 0.03; *F*(17,128) = 2.14, *p* = 0.009, respectively]. However, *post hoc* analyses did not indicate more improvement for children in the BGB EF training arm than for children in the placebo or waitlist arm, either for children above or for children below 10.5 years of age.

### Effect of the EF Training Program, Corrected for Gaming

Adding hours spent gaming outside school-hours to the mixed model analyses as a covariate showed that a significant interaction effect between treatment arm and time was now found for efficiency of the alerting network [*F*(4,129) = 8.85, *p* = 0.03]. *Post hoc* mixed model analyses showed larger improvement of efficiency of the alerting network for the placebo training arm than the EF training arm between baseline and first follow-up assessment. In addition, with time spent gaming in the model, a significant main effect of time was now found for the parent Emotional Symptoms scale of the SDQ [*F*(2,135) = 3.41, *p* = 0.04], suggesting less emotional problems over time. Furthermore, a significant main effect of time was found for self-perceived Behavioral Conduct [*F*(2,138) = 3.08, *p* = 0.049], indicating a reduction in behavioral problems over time. All other outcomes remained unchanged.

### Associations Between Gaming and Baseline Attentional, Behavioral and Emotional Functioning and Self-Perceived Competence

Hours spent gaming outside school-hours was significantly and inversely related to scores on both parent and teacher rated Prosocial Behavior on the SDQ, indicating that the more hours children spent gaming outside of school-hours, the less prosocial behavior parents and teachers reported (*r* = −0.23, *p* = 0.04; *r* = −0.25, *p* = 0.04, respectively). Furthermore, hours spent gaming outside of school-hours was significantly and positively related to scores on parent rated Hyperactivity on the SDQ (*r* = 0.23, *p* = 0.04), indicating that the more hours children spent gaming outside of school-hours, the more hyperactive behavior they showed. There were no other significant associations between gaming and any of the other baseline measures.

## Discussion

This study examined the effects of a computerized, game-formatted EF training program (BGB EF training program) on attentional, behavioral and emotional functioning and self-perceived competence of very preterm children in a double-blind, placebo and waitlist-controlled randomized trial. We first analyzed whether or not the intervention group showed improvements on the working memory, cognitive flexibility and inhibition tasks they trained during 12 weeks. Significant training effects were indeed found. Despite of this, results showed no positive effects of the BGB EF training program on any of the dependent measures.

In children with ADHD, promising effects of EF training programs on working memory were reported (Klingberg et al., 2005; Beck et al., 2010; Green et al., 2012; Hovik et al., 2013; Chacko et al., 2014; van der Oord et al., 2014; Dovis et al., 2015). However, in all of these studies, either a placebo or a waitlist-control group was included, but not both. Including a placebo condition enables to entangle specific and a-specific training effects, while including a waitlist-control group enables to entangle training effects (either specific or a-specific) from developmental effects and test-retest effects. In very preterm born adolescents aged 14–15 years, CWMT was shown to have positive effects on working memory and verbal learning (Lohaugen et al., 2011), however again only a non-intervention control group was included in that study, and no placebo control group, and the positive effects could thus reflect developmental or test–retest effects instead of effects of CWMT. Our results, without any beneficial effect of a computerized EF training program in very preterm children, are in line with the first randomized controlled trial on CWMT in very preterm children that did include a placebo control group, reporting no positive effects (Anderson et al., 2018). Literature on the effects of EF training programs is inconsistent at least and there is much debate on what effects EF training programs, including CWMT, actually have. Regarding the effects of working memory training on working memory performance, three meta-analyses have been performed, of which two conclude that EF training programs produce reliable improvements in both verbal and visuospatial working memory, with some evidence that the improvements in visuospatial working memory are maintained (Melby-Lervåg and Hulme, 2013; Aksayli et al., 2019). However, the third has theoretical arguments why simple span tasks are not a good measure for working memory improvement following CWMT and concludes that some studies using complex span tasks do and some studies do not find working memory improvements following CWMT (Shipstead et al., 2012). Regarding the effects of working memory training on other, untrained functions, these meta-analyses all three concluded that there was no evidence for generalization of working memory improvement to other domains (Shipstead et al., 2012; Melby-Lervåg and Hulme, 2013; Aksayli et al., 2019). Only one meta-analysis, performed by the research group involved in the development of CWMT (Spencer-Smith and Klingberg, 2016), concluded that CWMT has significant positive effects on inattention in daily life. However, comments on this study by Dovis et al. (2015a, b), have made arguments as to why these conclusions are controversial. In short, they state that: (1) there were coding errors in the initial meta-analysis, and after correction of these coding errors, effects of CWMT were no longer significant for several subgroup analyses, including for studies using an active or non-adaptive control group and for studies using a specific measure of inattention in daily life, (2) that differences between CWMT and control groups were analyzed without taking into account pre-test ratings of inattention, thus making it impossible to interpret which group benefits or improves most, or if there is any benefit or improvement at all and (3) that with correction for publication bias, the overall effect of CWMT on inattention was no longer significant, and that the reasons the authors of the meta-analysis provide for not correcting for publication bias are not supported by the literature.

The current study did not find positive effects of the BGB EF training program on attentional, behavioral and emotional functioning and self-perceived competence. Furthermore, meta-analyses have indicated no positive effects of the CWMT program for untrained functions. These results may be interpreted as game-based EF training being inadequate. However, as reported in the most recent meta-analysis on CWMT studies, this training induces moderate improvements in performance on memory tasks that are not included in the training or related to the trained tasks. This suggests that game-based EF training programs actually are able to improve working memory task performance, but that this improvement does not generalize to other functions. This could suggest that the game-based EF training programs need adjustments before they are capable to induce generalization of the trained functions to untrained functions. It could also suggest that the associations between EF deficits and problems in attentional, behavioral and emotional functioning that are commonly found (Nadeau et al., 2001; Taylor et al., 2006; Mulder et al., 2010, 2011; de Kieviet et al., 2012; Loe et al., 2012; Aarnoudse-Moens et al., 2013; Alduncin et al., 2014) are very complex, and that improvements in EFs alone do not directly lead to improvements in attentional, behavioral and emotional functioning. Furthermore, there may be limits to the plasticity of the brain of very preterm children, which may influence the extent to which game-based EF training leads to improvements in trained and untrained functions. Last, very preterm birth does not just influence the development of the child itself, but also has an impact on family functioning and parents’ functioning (Treyvaud, 2014) and subsequently parent-child interactions (Potharst et al., 2012). In 5-year-olds, mothers of very preterm children were less supportive of their children’s autonomy and interfered more often with their children’s autonomy than mothers of term born children (Potharst et al., 2012). In the setting of game-based EF training, this may lead to more negative interactions with the child about planning or execution of the training sessions, which in turn could lead to children being less motivated about the training. This may have negatively affected the extent to which children profit from the training.

The current study included children with a wide age range, including both children and adolescents (ages 8 years up to and including 12 years). As adolescence is a time in which significant neural, cognitive, behavioral and emotional changes take place (Spear, 2000; Yurgelun-Todd, 2007; Casey et al., 2008), effects of the BGB EF training program may differ depending on the ages studied. However, our analyses involving three-way interactions between treatment arm, time and age (above or below 10.5 years) showed that for almost all outcome measures, there was no differential effect of treatment arm over time between children above and below 10.5 years of age. Furthermore, for the two outcome measures for which there was a significant three-way interaction, there were no indications that the BGB EF training induced more improvement in either children above or below 10.5 years of age when compared to the placebo training and waitlist arm.

The interaction-effect for alerting network efficiency approached significance, and after time spent gaming before the intervention was taken into account, this interaction-effect became significant. However, for both, *post hoc* analyses showed that these interaction-effects were not indicative of larger improvements of alerting network efficiency in the EF training arm.

Significant improvements over time, regardless of treatment arm, were found for efficiency of the alerting and executive attention networks and for self-perceived competence in the domains of scholastics and athletics. After correction for time spent gaming before the intervention, there were also significant improvements over time for self-perceived behavioral conduct and parent-rated emotional symptoms. No negative changes over time were found. These improvements over time could be a sign of spontaneous recovery or regression to the mean. We also cannot exclude the explanation that this may be a Hawthorne effect, in which the effect of participating in research is reflected in a decrease in problems.

Our exploratory analyses revealed no large differences in outcomes of the analyses when these were adjusted for the time spent gaming outside school-hours. The small differences in outcomes when time spent gaming is adjusted for, may suggest that exposure to gaming at forehand does not influence the degree to which an EF training program as BGB may be effective.

Further analyses revealed that more time spent gaming outside school-hours at baseline assessment, was associated with more parent-rated hyperactive behavior and less prosocial behavioral according to both parents and teachers. Correlation obviously does not imply causation. Either way, our findings may suggest that if a computerized intervention is prescribed, it must be done in a healthy way, explaining child and parents that restrictions in time must be taken into account. For example, the American Academy of Pediatrics recommends that children have 2 h or less of sedentary screen time daily and that media-free times with the family and media-free locations in homes should be designated (Council on Communications and Media, 2016).

Is there still a future for EF training programs, or should focus shift away and focus on other promising interventions? The fact that improvements on the training tasks within the BGB EF training program took place, but no effects on the same EFs measured at follow-up assessments was found, suggests that improvement in the EFs was not just EF-specific, but also task-specific. From the skill learning field, it is known that transfer of learning from a trained task to even highly similar untrained tasks is generally the exception rather than the rule (Green and Bavelier, 2008). Training paradigms where more general learning has been established, are typically more complex and more ecologically valid, corresponding to real-life experiences (Green and Bavelier, 2008). One of the key factors in ensuring more general learning is variability in tasks and input (Green and Bavelier, 2008). In the BGB EF training program, only one EF is trained at a time and there is little correspondence to real-life experiences. For working memory, there is variability in task instructions and difficulty level, but not in the context in which the training task is performed or in what kind of working memory is trained (only visuospatial working memory, not verbal working memory). For inhibition and cognitive flexibility training, there is variability in difficulty level, but not in task instructions, context in which the training is performed or in the manner in which inhibition or cognitive flexibility is trained. Furthermore, for inhibition, only response inhibition is trained, while there are several other kinds of inhibition as well (Nigg, 2000). For CWMT, most of these arguments also apply; although several different working memory tasks are trained, there is little correspondence to real-life experiences and only one EF is trained at a time. Before abandoning the field of EF training programs, more ecologically valid EF training programs should be investigated for effectivity in improving EF and generalization of EF improvements to other functions such as attention. Focus could also shift to other promising interventions. Several activities seem to improve EFs in children in the general population, including traditional martial arts, aerobics, yoga, mindfulness, and several school curricula (Diamond, 2012). It has been suggested that especially interventions that address both EFs and children’s emotional, social and character development are effective (Diamond, 2012). Furthermore, two meta-analyses have shown that acute and longitudinal physical activity has positive effects on EF, attention and academic performance in children in the general population (Verburgh et al., 2014; de Greeff et al., 2018). Interventions as mentioned above have not yet been investigated in the very preterm population and thus should be subject of further research.

### Strengths and Limitations

Strengths of the current study are the incorporation of both a placebo training- and a waitlist-control arm, the use of intention-to-treat analyses, the objective measure of attentional functioning (efficiency of attention networks), the comprehensive assessment of behavioral and emotional functioning by both parents and teachers, and the assessment of both direct and longer-term effects. A limitation is that we failed to achieve our calculated sample, however, differences over time between groups were small and not clinically meaningful. Another limitation is the relatively high number of missing teacher SDQ questionnaires, however, as results on these measures are highly similar to results on the other outcome measures, we expect that a lower number of missing questionnaires would not have led to different results. As also in other studies using questionnaires (Simons et al., 2019), response rate on the CBCL in our study was low and possibly biased toward families of higher socio-economic status. Last, children with severe neonatal complications (IVH grade III or IV) were not excluded if they met inclusion criteria, which could have increased variability within the sample. However, sensitivity analyses including only children without severe neonatal complications were performed and results remained essentially unchanged.

## Conclusion

A computerized, game-formatted EF training program does not improve performance measures of attention, parent- or teacher rated behavioral and emotional functioning or self-perceived competence in very preterm children.

## Data Availability

The datasets generated for this study are available on request to the corresponding author.

## Ethics Statement

This study was approved by the Medical Ethical Committee of Amsterdam University Medical Centers and University Medical Center Utrecht. Written informed consent was obtained from the parents of all participants. Written informed consent was also obtained from participants aged >11 years.

## Author Contributions

CH contributed to the conceptualization, design, and methodology of the study, responsible for the outcome assessments and data collection, carried out the data analyses and interpretation, and wrote the manuscript. CA-M contributed to the conceptualization and design of the study, funding acquisition, data analysis methodology and data interpretation, overall supervision, and reviewed and revised the manuscript. AW-L contributed to the conceptualization and design of the study, funding acquisition, data interpretation, overall supervision, and reviewed and revised the manuscript. AL and CK-E contributed to the resources (participants), supervision in one of the Medical Centers, and reviewed and revised the manuscript. AK contributed to the conceptualization, design, and methodology of the study, data interpretation, overall supervision, and reviewed and revised the manuscript. JO contributed to the conceptualization, design, and methodology of the study, funding acquisition, data analysis methodology and data interpretation, overall supervision, and reviewed and revised the manuscript.

## Conflict of Interest Statement

The authors declare that the research was conducted in the absence of any commercial or financial relationships that could be construed as a potential conflict of interest.

## References

[B1] Aarnoudse-Moens C. S. H. Twilhaar E. S. Oosterlaan J. van Veen H. G. Prins P. J. M. van Kaam A. (2018). Executive function computerized training in very preterm-born children: a pilot study. *Games Health J.* 7 175–181. 10.1089/g4h.2017.0038 29641289

[B2] Aarnoudse-Moens C. S. H. Weisglas-Kuperus N. Duivenvoorden H. J. van Goudoever J. B. Oosterlaan J. (2013). Executive function and IQ predict mathematical and attention problems in very preterm children. *PLoS One* 8:e55994. 10.1371/journal.pone.0055994 23390558 PMC3563540

[B3] Aarnoudse-Moens C. S. H. Weisglas-Kuperus N. van Goudoever J. B. Oosterlaan J. (2009). Meta-analysis of neurobehavioral outcomes in very preterm and/or very low birth weight children. *Pediatrics* 124 717–728. 10.1542/peds.2008-2816 19651588

[B4] Aksayli N. D. Sala G. Gobet F. (2019). The cognitive and academic benefits of Cogmed: a meta-analysis. *Educ. Res. Rev*. 27 229–243. 10.1080/21622965.2013.875314 25010082

[B5] Alduncin N. Huffman L. C. Feldman H. M. Loe I. M. (2014). Executive function is associated with social competence in preschool-aged children born preterm or full term. *Early Hum. Dev.* 90 299–306. 10.1016/j.earlhumdev.2014.02.011 24661446 PMC4240273

[B6] Allotey J. Zamora J. Cheong-See F. Kalidindi M. Arroyo-Manzano D. Asztalos E. (2017). Cognitive, motor, behavioural and academic performances of children born preterm: a meta-analysis and systematic review involving 64 061 children. *BJOG* 125 16–25. 10.1111/1471-0528.14832 29024294

[B7] Anderson P. J. Doyle L. W. The Victorian Infant Collaborative Study Group. (2003). Neurobehavioral outcomes of school-age children born extremely low birth weight or very preterm in the 1990s. *JAMA* 289 3264–3272. 12824207 10.1001/jama.289.24.3264

[B8] Anderson P. J. Lee K. J. Roberts G. Spencer-Smith M. M. Thompson D. K. Seal M. L. (2018). Long-term academic functioning following cogmed working memory training for children born extremely preterm: a randomized controlled trial. *J. Pediatr.* 202 92.e4–97.e4. 10.1016/j.jpeds.2018.07.003 30177350

[B9] Aylward G. P. (2014). Neurodevelopmental outcomes of infants born prematurely. *J. Dev. Behav. Pediatr.* 35 394–407. 10.1097/01.DBP.0000452240.39511.d4 25007063

[B10] Barnes J. J. Nobre A. C. Woolrich M. W. Baker K. Astle D. E. (2016). Training working memory in childhood enhances coupling between frontoparietal control network and task-related regions. *J. Neurosci.* 36 9001–9011. 10.1523/JNEUROSCI.0101-16.2016 27559180 PMC4995310

[B11] Beck S. J. Hanson C. A. Puffenberger S. S. Benninger K. L. Benninger W. B. (2010). A controlled trial of working memory training for children and adolescents with ADHD. *J. Clin. Child Adolesc. Psychol.* 39 825–836. 10.1080/15374416.2010.517162 21058129

[B12] Bhutta A. T. Cleves M. A. Casey P. H. Cradock M. M. Anand K. J. (2002). Cognitive and behavioral outcomes of school-aged children who were born preterm: a meta-analysis. *JAMA* 288 728–737. 12169077 10.1001/jama.288.6.728

[B13] Blencowe H. Cousens S. Chou D. Oestergaard M. Say L. Moller A.-B. (2013). Born too soon: the global epidemiology of 15 million preterm births. *Reprod. Health* 10(Suppl. 1), S2–S2. 10.1186/1742-4755-10-S1-S2 24625129 PMC3828585

[B14] Carlson S. M. Wang T. S. (2007). Inhibitory control and emotion regulation in preschool children. *Cogn. Dev.* 22 489–510. 10.1016/j.cogdev.2007.08.002

[B15] Casey B. J. Getz S. Galvan A. (2008). The adolescent brain. *Dev. Rev.* 28 62–77. 10.1016/j.dr.2007.08.003 18688292 PMC2500212

[B16] Chacko A. Bedard A. Marks D. Feirsen N. Uderman J. Chimiklis A. (2014). A randomized clinical trial of Cogmed working memory training in school-age children with ADHD: a replication in a diverse sample using a control condition. *J. Child Psychol. Psychiatry* 55 247–255. 10.1111/jcpp.12146 24117656 PMC3944087

[B17] Chorne N. Leonard C. Piecuch R. Clyman R. I. (2007). Patent Ductus arteriosus and its treatment as risk factors for neonatal and neurodevelopmental morbidity. *Pediatrics* 119 1165–1174. 10.1542/peds.2006-3124 17545385

[B18] Council on Communications and Media (2016). Media use in school-aged children and adolescents. *Pediatrics* 138:e20162592. 10.1542/peds.2016-2592 27940794

[B19] de Greeff J. W. Bosker R. J. Oosterlaan J. Visscher C. Hartman E. (2018). Effects of physical activity on executive functions, attention and academic performance in preadolescent children: a meta-analysis. *J. Sci. Med. Sport* 21 501–507. 10.1016/j.jsams.2017.09.595 29054748

[B20] de Kieviet J. F. van Elburg R. M. Lafeber H. N. Oosterlaan J. (2012). Attention problems of very preterm children compared with age-matched term controls at school-age. *J. Pediatr.* 161 824–829. 10.1016/j.jpeds.2012.05.010 22704248

[B21] de Vries M. Prins P. J. Schmand B. A. Geurts H. M. (2015). Working memory and cognitive flexibility-training for children with an autism spectrum disorder: a randomized controlled trial. *J. Child Psychol. Psychiatry* 56 566–576. 10.1111/jcpp.12324 25256627

[B22] Delnord M. Hindori-Mohangoo A. D. Smith L. K. Szamotulska K. Richards J. L. Deb-Rinker P. (2017). Variations in very preterm birth rates in 30 high-income countries: are valid international comparisons possible using routine data? *BJOG* 124 785–794. 10.1111/1471-0528.14273 27613083 PMC5346062

[B23] Diamond A. (2012). Activities and programs that improve children’s executive functions. *Curr. Dir. Psychol. Sci.* 21 335–341. 10.1177/0963721412453722 25328287 PMC4200392

[B24] Dice J. E. Bhatia J. (2007). Patent ductus arteriosus: an overview. *J. Pediatr. Pharmacol. Therapeut.* 12 138–146. 10.5863/1551-6776-12.3.138 23055849 PMC3462096

[B25] Dovis S. Agelink van Rentergem J. Huizenga H. (2015a). Does cogmed working memory training really improve inattention in daily life? A reanalysis. *PLoS One* 10:e0119522. 10.1371/journal.pone.0119522 27875585 PMC5119832

[B26] Dovis S. Agelink van Rentergem J. Huizenga H. (2015b). Response to the correction by Spencer-Smith and Klingberg: unaddressed concerns. *PLoS One* 10:e0119522. 10.1371/journal.pone.0119522 27875585 PMC5119832

[B27] Dovis S. Van der Oord S. Wiers R. W. Prins P. J. (2015). Improving executive functioning in children with ADHD: training multiple executive functions within the context of a computer game. A randomized double-blind placebo controlled trial. *PLoS One* 10:e0121651. 10.1371/journal.pone.0121651 25844638 PMC4386826

[B28] Ghosh D. Vogt A. (2012). Outliers: an evaluation of methodologies. *Joint Stat. Meet.* 3455–3460.

[B29] Green C. S. Bavelier D. (2008). Exercising your brain: a review of human brain plasticity and training-induced learning. *Psychol. Aging* 23 692–701. 10.1037/a0014345 19140641 PMC2896818

[B30] Green C. T. Long D. L. Green D. Iosif A.-M. Dixon J. F. Miller M. R. (2012). Will working memory training generalize to improve off-task behavior in children with attention-deficit/hyperactivity disorder? *J. Neurother.* 9 639–648. 10.1007/s13311-012-0124-y 22752960 PMC3441930

[B31] Hovik K. T. Saunes B. K. Aarlien A. K. Egeland J. (2013). RCT of working memory training in ADHD: long-term near-transfer effects. *PLoS One* 8:e80561. 10.1371/journal.pone.0080561 24352414 PMC3857172

[B32] Hudziak J. J. Copeland W. Stanger C. Wadsworth M. (2004). Screening for DSM-IV externalizing disorders with the child behavior checklist: a receiver-operating characteristic analysis. *J. Child Psychol. Psychiatry* 45 1299–1307. 10.1111/j.1469-7610.2004.00314.x 15335349

[B33] IBM (2017). *IBM SPSS Statistics for Windows, Version 25.0.* Armonk, NY: IBM Corp.

[B34] Klingberg T. Fernell E. Olesen P. J. Johnson M. Gustafsson P. Dahlstrom K. (2005). Computerized training of working memory in children with ADHD–a randomized, controlled trial. *J. Am. Acad. Child Adolesc. Psychiatry* 44 177–186. 10.1097/00004583-200502000-00010 15689731

[B35] Loe I. M. Lee E. S. Luna B. Feldman H. M. (2012). Executive function skills are associated with reading and parent-rated child function in children born prematurely. *Early Hum. Dev.* 88 111–118. 10.1016/j.earlhumdev.2011.07.018 21849240 PMC3660611

[B36] Lohaugen G. C. Antonsen I. Haberg A. Gramstad A. Vik T. Brubakk A. M. (2011). Computerized working memory training improves function in adolescents born at extremely low birth weight. *J. Pediatr.* 158 555.e4–561.e4. 10.1016/j.jpeds.2010.09.060 21130467

[B37] Melby-Lervåg M. Hulme C. (2013). Is working memory training effective? A meta-analytic review. *Dev. Psychol.* 49 270–291. 10.1037/a0028228 22612437

[B38] Metzler-Baddeley C. Caeyenberghs K. Foley S. Jones D. K. (2016). Task complexity and location specific changes of cortical thickness in executive and salience networks after working memory training. *J. Neuroimage* 130 48–62. 10.1016/j.neuroimage.2016.01.007 26806288 PMC4819728

[B39] Metzler-Baddeley C. Foley S. de Santis S. Charron C. Hampshire A. Caeyenberghs K. (2017). Dynamics of white matter plasticity underlying working memory training: multimodal evidence from diffusion MRI and relaxometry. *J. Cogn. Neurosci.* 29 1509–1520. 10.1162/jocn_a_01127 28358656 PMC5881889

[B40] Mulder H. Pitchford N. J. Hagger M. S. Marlow N. (2009). Development of executive function and attention in preterm children: a systematic review. *Dev. Neuropsychol.* 34 393–421. 10.1080/87565640902964524 20183707

[B41] Mulder H. Pitchford N. J. Marlow N. (2010). Processing speed and working memory underlie academic attainment in very preterm children. *Arch. Dis. Child Fetal Neonatal Ed.* 95 F267–F272. 10.1136/adc.2009.167965 20488865

[B42] Mulder H. Pitchford N. J. Marlow N. (2011). Inattentive behaviour is associated with poor working memory and slow processing speed in very pre-term children in middle childhood. *Br. J. Educ. Psychol.* 81(Pt 1), 147–160. 10.1348/000709910x505527 21391967

[B43] Nadeau L. Boivin M. Tessier R. Lefebvre F. Robaey P. (2001). Mediators of behavioral problems in 7-year-old children born after 24 to 28 weeks of gestation. *J. Dev. Behav. Pediatr.* 22 1–10. 10.1097/00004703-200102000-00001 11265917

[B44] Nigg J. T. (2000). On inhibition/disinhibition in developmental psychopathology: views from cognitive and personality psychology and a working inhibition taxonomy. *Psychol. Bull.* 126 220–246. 10.1037//0033-2909.126.2.220 10748641

[B45] Oosterlaan J. Scheres A. Sergeant J. A. (2005). Which executive functioning deficits are associated with AD/HD, ODD/CD and comorbid AD/HD+ ODD/CD? *J. Abnorm. Child Psychol.* 33 69–85. 10.1007/s10802-005-0935-y 15759592

[B46] Ozonoff S. Pennington B. F. Rogers S. J. (1991). Executive function deficits in high-functioning autistic individuals: relationship to theory of mind. *J. Child Psychol. Psychiatry* 32 1081–1105. 10.1111/j.1469-7610.1991.tb00351.x 1787138

[B47] Pennington B. F. Ozonoff S. (1996). Executive functions and developmental psychopathology. *J. Child Psychol. Psychiatry* 37 51–87. 10.1111/j.1469-7610.1996.tb01380.x 8655658

[B48] Potharst E. S. Schuengel C. Last B. F. van Wassenaer A. G. Kok J. H. Houtzager B. A. (2012). Difference in mother-child interaction between preterm- and term-born preschoolers with and without disabilities. *Acta Paediatr.* 101 597–603. 10.1111/j.1651-2227.2012.02599.x 22536811

[B49] Prins P. J. Brink E. T. Dovis S. Ponsioen A. Geurts H. M. De Vries M. (2013). “Braingame Brian”: toward an executive function training program with game elements for children with ADHD and cognitive control problems. *Games Health J.* 2 44–49. 10.1089/g4h.2013.0004 26196554

[B50] Riggs N. R. Jahromi L. B. Razza R. P. Dillworth-Bart J. E. Mueller U. (2006). Executive function and the promotion of social–emotional competence. *J. Appl. Dev. Psychol.* 27 300–309. 10.1016/j.jecp.2010.08.004 20828709 PMC3016464

[B51] Ritchie K. Bora S. Woodward L. J. (2015). Social development of children born very preterm: a systematic review. *Dev. Med. Child Neurol.* 57 899–918. 10.1111/dmcn.12783 25914112

[B52] Rueda M. R. Fan J. McCandliss B. D. Halparin J. D. Gruber D. B. Lercari L. P. (2004). Development of attentional networks in childhood. *J. Neuropsychol.* 42 1029–1040. 10.1016/j.neuropsychologia.2003.12.012 15093142

[B53] Sattler J. M. (1992). *Assessment of Children: WISC—III and WPPSI—R Supplement.* San Diego, CA: Jerome M. Sattler.

[B54] Sergeant J. A. Geurts H. Oosterlaan J. (2002). How specific is a deficit of executive functioning for attention-deficit/hyperactivity disorder? *Behav. Brain Res.* 130 3–28. 10.1016/s0166-4328(01)00430-2 11864714

[B55] Shipstead Z. Hicks K. L. Engle R. W. (2012). Cogmed working memory training: does the evidence support the claims? *J. Appl. Res. Mem. Cogn.* 1 185–193. 10.1016/j.jarmac.2012.06.003

[B56] Simons N. E. van de Beek C. van der Lee J. H. Opmeer B. C. van Wassenaer-Leemhuis A. G. van Baar A. L. (2019). Child outcomes after placement of a cervical pessary in women with a multiple pregnancy: a 4-year follow-up of the ProTWIN trial. *Acta Obstet. Gynecol. Scand.* 10.1111/aogs.13630 [Epub ahead of print]. 31032879 PMC6900136

[B57] Spear L. P. (2000). Neurobehavioral changes in adolescence. *Curr. Dir. Psychol. Sci.* 9 111–114. 10.1111/1467-8721.00072

[B58] Spencer-Smith M. Klingberg T. (2016). Correction: benefits of a working memory training program for inattention in daily life: a systematic review and meta-analysis. *PLoS One* 11:e0167373. 10.1371/journal.pone.0167373 27875585 PMC5119832

[B59] Stevens M. C. Gaynor A. Bessette K. L. Pearlson G. D. (2016). A preliminary study of the effects of working memory training on brain function. *Brain Imaging Behav.* 10 387–407. 10.1007/s11682-015-9416-2 26138580 PMC4698365

[B60] Taylor H. G. Klein N. Drotar D. Schluchter M. Hack M. (2006). Consequences and risks of <1000-g birth weight for neuropsychological skills, achievement, and adaptive functioning. *J. Dev. Behav. Pediatr.* 27 459–469. 10.1097/00004703-200612000-00002 17164618

[B61] Treyvaud K. (2014). Parent and family outcomes following very preterm or very low birth weight birth: a review. *Semin. Fetal Neonatal Med.* 19 131–135. 10.1016/j.siny.2013.10.008 24252709

[B62] Twilhaar E. S. de Kieviet J. F. Aarnoudse-Moens C. S. van Elburg R. M. Oosterlaan J. (2017). Academic performance of children born preterm: a meta-analysis and meta-regression. *Arch. Dis. Child Fetal Neonatal Ed.* 103 F322–F330. 10.1136/archdischild-2017-312916 28847871 PMC6047144

[B63] Twisk J. W. (2013). *Applied Longitudinal Data Analysis for Epidemiology: A Practical Guide.* Cambridge, MA: University Press.

[B64] van der Oord S. Ponsioen A. J. Geurts H. M. Ten Brink E. L. Prins P. J. (2014). A pilot study of the efficacy of a computerized executive functioning remediation training with game elements for children with ADHD in an outpatient setting: outcome on parent- and teacher-rated executive functioning and ADHD behavior. *J. Atten. Disord.* 18 699–712. 10.1177/1087054712453167 22879577

[B65] van Houdt C. A. Oosterlaan J. van Wassenaer-Leemhuis A. G. van Kaam A. H. Aarnoudse-Moens C. S. H. (2019). Executive function deficits in children born preterm or at low birthweight: a meta-analysis. *Dev. Med. Child Neurol.* 61 1015–1024. 10.1111/dmcn.14213 30945271 PMC6850293

[B66] van Widenfelt B. M. Goedhart A. W. Treffers P. D. Goodman R. (2003). Dutch version of the strengths and difficulties questionnaire (SDQ). *Eur. Child Adolesc. Psychiatry* 12 281–289. 10.1007/s00787-003-0341-3 14689260

[B67] Veerman J. W. Straathof M. Treffers A. Van den Bergh B. Ten Brink T. (1997). *Handleiding Competentiebelevingsschaal Voor Kinderen (CBSK).* Lisse: Swets & Zeitlinger.

[B68] Verburgh L. Königs M. Scherder E. J. Oosterlaan J. (2014). Physical exercise and executive functions in preadolescent children, adolescents and young adults: a meta-analysis. *Br. J. Sports Med.* 48 973–979. 10.1136/bjsports-2012-091441 23467962

[B69] Verhulst F. C. Van der Ende J. (2013). *Handleiding ASEBA-Vragenlijsten voor Leeftijden 6 t/m 18 Jaar: CBCL/6-18, YSR en TRF.* Rotterdam: ASEBA Nederland.

[B70] Willcutt E. G. Doyle A. E. Nigg J. T. Faraone S. V. Pennington B. F. (2005). Validity of the executive function theory of attention-deficit/hyperactivity disorder: a meta-analytic review. *Biol. Psychiatry* 57 1336–1346. 10.1016/j.biopsych.2005.02.006 15950006

[B71] Yurgelun-Todd D. (2007). Emotional and cognitive changes during adolescence. *Curr. Opin. Neurobiol.* 17 251–257. 10.1016/j.conb.2007.03.009 17383865

